# Research Progress on the Relationship Between the Intestinal Barrier and Macrophages

**DOI:** 10.3390/cimb47100813

**Published:** 2025-10-02

**Authors:** Shan Ma, Kecheng Zhu, Yan Liu, Jizhuang Wang

**Affiliations:** 1 Department of Burn, Ruijin Hospital, Shanghai Jiao Tong University School of Medicine, Shanghai 200025, China; 2 Department of Endocrine and Metabolic Diseases, Shanghai Institute of Endocrine and Metabolic Diseases, Ruijin Hospital School of Medicine, Shanghai Jiao Tong University, Shanghai 200025, China

**Keywords:** intestinal barrier, macrophages, metabolic crosstalk

## Abstract

The intestinal barrier serves as a crucial defense in the body, and a compromised integrity can lead to systemic health issues. Intestinal macrophages, as key components of the intestinal immune system, have a profound influence on intestinal homeostasis. However, the complex mechanisms underlying interactions between the intestinal barrier and intestinal macrophages remain incompletely elucidated. This review systematically summarizes the bidirectional regulatory relationship between them. It specifically focuses on how the intestinal barrier impacts the phenotype, function, and heterogeneity of macrophages while concurrently analyzing the pivotal role of macrophages in maintaining and repairing intestinal barrier function. Furthermore, this review provides an in-depth analysis of the critical influence of this interaction on the pathogenesis and progression of various intestinal disorders and systemic diseases. By synthesizing and summarizing current research advances, this review aims to enhance the understanding of the regulatory mechanisms underlying intestinal immune homeostasis and to lay a theoretical foundation for developing novel strategies for the prevention and treatment of related diseases.

## 1. Introduction

The intestinal barrier, comprising mechanical, biological, immune, and chemical components, serves as the primary defense against pathogens and maintains internal homeostasis [1]. Its integrity relies on the dynamic crosstalk between intestinal barrier components and innate immune cells, particularly macrophages [2]. However, the spatiotemporal heterogeneity of intestinal macrophage subsets and their precise interplay with intestinal barrier components remain poorly characterized. This knowledge gap impedes the development of targeted therapies for barrier dysfunction diseases like inflammatory bowel disease (IBD) and sepsis. This review synthesizes recent evidence on the interaction between macrophages and the intestinal barrier.

## 2. Literature Search and Screening Strategy

This review systematically investigates the crosstalk between intestinal macrophages and the intestinal barrier. Following PRISMA guidelines, a comprehensive literature search was performed in May 2025 using PubMed/MEDLINE and Web of Science Core Collection. The search strategy, based on the PICOS framework and incorporating concepts such as “intestinal macrophages” and “intestinal barrier”, employed relevant synonyms and Boolean operators. The searches were limited to English-language articles published between January 2000 and May 2025.

Records were imported into EndNote 20 for duplicate removal. Two reviewers independently screened titles and abstracts against the inclusion criteria: original research articles and reviews examining molecular mechanisms or functional outcomes of macrophage–barrier cell interactions. Exclusions included non-English publications, studies focusing solely on one cell type, or those with irrelevant research foci. Full texts of selected articles were assessed for final inclusion.

Data extraction focused on study characteristics, experimental models, cell types, mechanisms, and key findings. A narrative synthesis approach was adopted to summarize the evidence, with due consideration given to the strengths and limitations of the study models in the interpretation of results.

## 3. Overview of Intestinal Barrier

**Figure 1:**
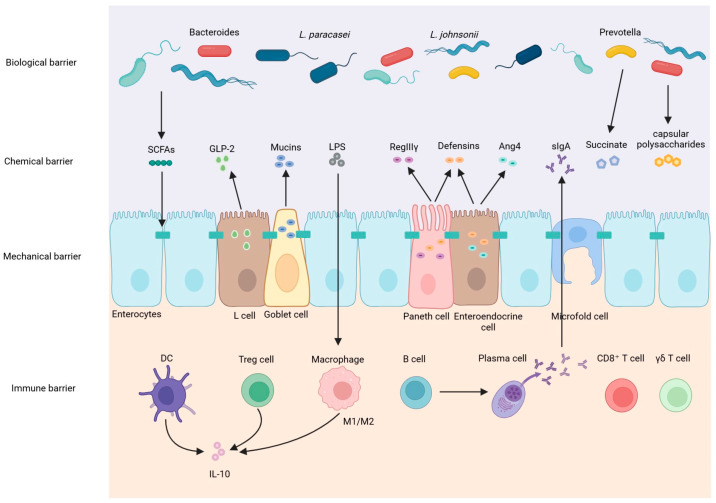
Overview of intestinal barrier.

The intestinal barrier is composed of mechanical, biological, immune, and chemical components. The mechanical barrier comprises a single layer of intestinal epithelial cells (IECs) interconnected by tight junction proteins such as claudin, occludin, and Zona Occludens 1 (ZO-1), forming a selectively permeable interface. Positioned at the interface between intestinal symbionts and macrophages, intestinal epithelial cells include specialized subtypes such as enterocytes, Paneth cells, goblet cells, enteroendocrine cells, and microfold cells. These IECs undergo complete renewal every 3–5 days through crypt stem cell differentiation, while goblet cell-derived mucus layers provide a physical shield against pathogens [3]. Tight junction integrity within the epithelial barrier is dynamically controlled by functional proteins. Dysregulated phosphorylation of ZO-1 and occludin directly compromises barrier function, resulting in intestinal hyperpermeability (“leaky gut”) [4]. Glucagon-like peptide 2 (GLP-2) augments intestinal barrier integrity through dual mechanisms: expanding stem cell populations to drive epithelial proliferation while suppressing NLRP3 inflammasome activation, thereby bolstering tight junctions and curtailing LPS translocation into the circulation [5] (Figure 1).

The biological barrier is a vital defense formed by the trillions of commensal microbes (bacteria, viruses, fungi) and their metabolites living in the intestines [6]. Beneficial microbes block harmful invaders by competing for space and food, producing natural antimicrobials, and maintaining a protective low-oxygen environment. Crucially, these beneficial microbes support the intestinal lining itself. Through dietary fiber fermentation, intestinal microbiota produce short-chain fatty acids (SCFAs) such as butyrate, which strengthen intestinal barrier integrity by upregulating tight junction proteins while inhibiting pathogen colonization by such as *Salmonella* [7]. Beneficial microbes also help digest complex carbs and make essential vitamins like B vitamins and K. Furthermore, this microbial community is essential for training and regulating the immune system. Regarding microbiota–barrier interactions, capsular polysaccharides from *Bacteroides* spp. sustain the mucus layer through Toll Like Receptor 2 (TLR2) activation, and succinate produced by *Prevotella* spp. facilitates damage repair via the succinate receptor antagonist 1 (SUCNR1) receptor . The microbiota teaches the body to distinguish friend from foe, promotes tolerance to prevent harmful inflammation, and primes defenses against pathogens. Its influence extends beyond the intestine, impacting metabolism, mood, brain function, and overall health via pathways like the gut–brain axis (Figure 1).

The immune barrier serves as the core defense mechanism. Its function depends on gut-associated lymphoid tissue (GALT), encompassing Peyer’s patches, isolated lymphoid follicles, and mesenteric lymph nodes. The immune barrier operates via GALT, where lamina propria macrophages maintain epithelial integrity through apoptotic cell clearance [8], while CD4^+^ Treg cells secrete IL-10 to suppress hyperinflammation [9]. Specialized M cells within this system are responsible for the active uptake of antigens from the intestinal lumen. Intraepithelial lymphocytes (IELs) such as CD8^+^ T cells and γδ T cells act as frontline sentinels; within the lamina propria, dendritic cells are key antigen-presenting cells, and various T cell subsets along with antibody-producing B cells/plasma cells collectively form a dynamic defense network. In addition, the crucial effector molecule is secretory immunoglobulin A (sIgA). Produced by plasma cells and transported into the intestinal lumen, sIgA neutralizes pathogens, prevents their adhesion, clears antigen complexes, and gently modulates microbial balance [10] (Figure 1). Critically, it accomplishes these functions without triggering excessive inflammation, making it essential for maintaining mucosal homeostasis.

The chemical barrier provides immediate defense. Gastric acid, digestive enzymes, and bile salts break down pathogens in the upper gastrointestinal tract. In the small intestine, Paneth cells and epithelial cells secrete various antimicrobial peptides (such as defensins, α-defensins, RegIIIγ, and lysozyme), which can directly lyse pathogens [11], while bile acids enhance chemical defense by regulating antimicrobial peptide expression via the Farnesoid X Receptor (FXR) receptors [12]. Goblet cell-derived mucin2 (MUC2) mucins forming a stratified mucus layer—an outer (microbiota-colonized) and an inner (sterile)—whose thickness is modulated by IL-22 [13] (Figure 1).

## 4. Overview of Intestinal Macrophages

Intestinal macrophages, as a central component of the gut immune barrier, engage in close and dynamic interactions with various intestinal barrier cells to collectively maintain mucosal homeostasis and host defense.

### 4.1. Origin and Development

The intestinal macrophage population maintains a dynamic equilibrium. Embryonic yolk sac-derived cells serve as the guardians of the physiological barrier, while the bone marrow monocyte-derived population acts as a double-edged sword of the inflammatory response [14,15].

A clear dual pattern links the developmental origin and functional regulation of intestinal macrophages. Under homeostasis, embryonically derived macrophages maintain tissue integrity through self-renewal. They exhibit an anti-inflammatory phenotype, characterized by high expression of CX3CR1, Tim-4, and LYVE-1, and secrete IL-10 and TGF-β to promote tolerance and clear apoptotic cells [14]. In contrast, during inflammation, bone marrow-derived Ly6C^+^CCR2^+^ monocytes are recruited and differentiate into pro-inflammatory macrophages [15]. These cells produce TNF-α, IL-6, IL-1β, IL-23, and iNOS to combat pathogens [16,17]. However, their sustained activation exacerbates tissue damage. This dual system enables a critical balance between immune defense and tolerance.

Early investigations established that intestinal CX3CR1^+^ macrophages originate predominantly from yolk sac-derived erythro-myeloid progenitors (EMPs) under homeostatic conditions [14]. Their development occurs independently of hematopoietic stem cells (HSCs) and requires no input from the transcription factor Myb [18]. Postnatally, these embryonically seeded macrophages self-renew locally through in situ proliferation. Characteristically, they exhibit high expression of the anti-inflammatory cytokine IL-10 and phagocytic receptor Tim-4, enabling them to mediate apoptotic cell clearance and sustain intestinal immune tolerance [14]. However, during inflammatory or injured states, bone marrow-derived Ly6C^+^CCR2^+^ monocytes are recruited into the intestine in large numbers where they differentiate into pro-inflammatory macrophages and replace the embryonic-derived population [15]. This switch in cellular origin leads to functional imbalance. Upon intestinal disruption, circulating Ly6C+CCR2^+^ monocytes rapidly recruit to injury sites, where they differentiate into pro-inflammatory macrophages. These cells secrete TNF-α and IL-6 [16,17], which enhance pathogen clearance but exacerbate tissue damage and impair repair processes. CCR2+ macrophage infiltration levels in IBD patient intestinal biopsies positively correlate with disease severity [15]. Inflammation drives aberrant macrophage differentiation into pro-inflammatory states, as exemplified by the transformation of Ly6ChiCX3CR1int monocytes [19]. These polarized macrophages exacerbate tissue damage—worsening enteritis, fibrosis, and epithelial injury—through IL-23 secretion [20].

### 4.2. Regional Specialization

Intestinal macrophages exhibit spatial heterogeneity in phenotype and marker expression, reflecting location-specific functional adaptations [5,6,21,22]. In the lamina propria, macrophages express CD68, CD11b, CD14, and CX3CR1. They comprise a homeostatic subset (Tim-4^+^ LYVE-1^+^ CD4^+^ in mice) that clears apoptotic cells and maintains immune tolerance via IL-10 secretion [21,22], and an inflammatory monocyte-derived subset (CCR2^+^ Ly6C^+^) that produces pro-inflammatory cytokines including TNF-α and IL-6 [23]. Adjacent to the epithelium, CX3CR1^h4^ macrophages extend transepithelial protrusions to sample luminal antigens, serving a critical role in immune surveillance [5,6]. Within the muscularis layers, macrophages express CD68, CX3CR1, and high levels of CD169, with a subset positive for LYVE-1. These cells interact closely with enteric neurons and glia to modulate intestinal motility and contribute to tissue repair [24].

Building on this developmental origin, regional specialization further defines macrophage functionality. CX3CR1^+^CD206^+^ macrophages in the small intestinal lamina propria maintain barrier integrity through continuous clearance of commensal antigens and cellular debris while suppressing hyperinflammation via IL-10 secretion [25]. In contrast, colonic macrophages contribute to microbiota regulation. Evidence suggests the presence of spatial heterogeneity in intestinal macrophage subsets, with ileal PTGER4+ and colonic CRIg+ subsets being notable examples [26]. These cells exert intestinal protective effects through the production of anti-inflammatory factors such as NADinduced immunoregulation [27] and through tissue repair functions mediated by non-canonical Nr4a1^+^ monocyte-derived repair macrophages [28]. Specialized macrophage subsets perform niche functions; for example, LGR5^+^ macrophages localize to crypt bases, secreting Wnt3a/R-spondin to activate intestinal stem cell β-catenin signaling and enhancing cell survival via E-cadherin contacts—mechanisms critical for post-injury regeneration [29].

## 5. How Intestinal Barrier Affects Macrophages

The interplay between intestinal barrier cells and macrophages is fundamental for intestinal homeostasis. This state critically depends on dynamic crosstalk between epithelial and immune cells, which encompasses structural, metabolic, and immunological interactions.

Within this network, the core cellular elements of the barrier include IECs, goblet cells, and Paneth cells. These barrier cells perform dual essential functions. Firstly, they prevent pathogen invasion. Secondly, they actively shape macrophage polarization states, partly through metabolite secretion, signaling mediators, and direct cellular contacts. This coordinated dialogue carefully balances inflammatory responses with regenerative processes. Disruption of this system directly contributes to the development of inflammatory pathologies like IBD. The oversimplified dichotomy between M1 and M2 phenotypes is increasingly viewed as an inadequate representation of the intricate and multifaceted nature of macrophage responses. We therefore use the terms “pro-inflammatory” and “anti-inflammatory” to replace the strict M1/M2 dichotomy, as these more accurately reflect the continuum and context-dependent nature of macrophage function.

As the primary physical barrier, IECs form a selectively permeable layer through tight junction proteins such as occludin and claudin-3. Claudin-3 deficiency increases permeability and the risk of colitis by promoting dysbiosis [11]. Damaged IECs release ATP, which activates the NLRP3 inflammasome in macrophages via Purinergic 2X7 (P2X7), as demonstrated in skin allograft models [30]. Mitochondrial dysfunction in IECs amplifies pro-inflammatory macrophages polarization through ROS-dependent NOX4 pathways in macrophages [31]. In IBD, impaired IEC mitochondrial respiration leads to succinate accumulation, activating macrophage SUCNR1 receptors to fuel IL-1β and IL-18 secretion, perpetuating an “inflammation-oxidative stress” cycle [7,32]. Notably, IEC-derived indole metabolites promote anti-inflammatory IL-10 secretion in macrophages via aryl hydrocarbon receptor (AhR) signaling [33], underscoring their dual role in immune regulation.

Goblet cells regulate macrophage activity through mucin secretion and chemokine signaling. Loss of MUC2, the primary mucus component, exposes the epithelium to microbiota, triggering IL-23/IL-17-driven inflammation via macrophage activation [34]. Conversely, MUC2 degradation products activate macrophage G protein-coupled receptor 109 A (GPR109A) receptors to enhance IL-10 production and anti-inflammatory macrophage polarization—a mechanism disrupted in IBD patients with goblet cell depletion [35,36]. Goblet cell-secreted CCL20 recruits CCR6^+^ immune cells [37],establishing mucosal immunity.

Paneth cells secrete RegIIIγ to spatially segregate microbiota from the epithelium [38]. Lysozyme-digested muramyl dipeptide (MDP) activates macrophage nucleotide-binding oligomerization domain 2 (NOD2) to enhance autophagy [39]. Dysfunctional Paneth cells with autophagy related protein 16 like protein 1 (ATG16L1) mutations disrupt autophagy, exacerbating inflammation [40]. Paneth cell antimicrobial peptide secretion is enhanced through the IL-23-IL-22 axis to defend against pathogenic invasion [41].

## 6. How Macrophages Affect Intestinal Barrier

LGR5^+^ macrophage subsets activate crypt stem cells through Wnt3a/R-spondin signaling to drive crypt regeneration [42]. Meanwhile, macrophages sense commensal bacterial signals through TLR2/4 and secrete IL-10 to inhibit hyperinflammation and maintain epithelial tight junction integrity [43]. Macrophages act as central immunological hubs. They integrate multiple critical processes: epithelial repair, neuromodulation, and systemic inflammation. This integration occurs through their release of diverse mediators, including metabolites, EVs, cytokines, and neurotrophic factors. CX3CR1^+^ macrophages induce tertiary lymphoid structures, while IL-10-dependent subsets coordinate colitis repair. This content is categorized and presented in Table 1.

In terms of barrier disruption, pro-inflammatory macrophages release TNF-α and IL-1β through the NOX4/ROS pathway and CCL3-mediated neutrophil recruitment, directly degrading tight junction proteins, while up-regulation of Claudin-2 forms abnormal pores to increase intestinal permeability, resulting in microbiota translocation and systemic inflammation. Barrier damage further weakens macrophage clearance, forming a microbiota–macrophage vicious circle. Dysbiosis components such as LPS continuously activates the macrophage TLR/NF-κB pathway through TLR4-MyD88 signaling [44] and promote intestinal leakage [44,45]. In pathological states such as IBD, macrophage dysfunction leads to overactivation of the IL-23/IL-17 pathway, which exacerbates barrier disruption and chronic inflammation [17,46]. Targeting macrophage-epithelial interactions such as anti-integrin α4β7 therapy) has shown potential to restore barrier function [47] (Table 1).

In terms of barrier protection, at the level of immune regulation, CD11c^+^ macrophages induce Treg differentiation through TGF-β and retinoic acid to prevent abnormal responses to commensal bacteria [48] (Table 1). Anti-inflammatory macrophages secrete IL-10 and activate the epithelial JAK1/STAT3 pathway [49,50,51] to promote tight junction protein synthesis. Key molecules such as LACC1 [51] enhance macrophage bacterial clearance and reduce microbiota translocation; Heme induces anti-inflammatory macrophage polarization to repair the barrier through both HO-1-dependent and independent pathways [50] (Table 2). In addition, probiotics such as L. paracasei and its extracellular polysaccharide [49] promote macrophage anti-inflammatory polarization by balancing Th17/Treg cells, thereby enhancing the mucus layer and tight junctions; GM-CSF [52] maintains macrophage homeostasis to support epithelial repair (Table 1).

### 6.1. Multiple Signaling Interact to Regulate Intestinal Barrier Function

Macrophage Tim-3 deficiency exacerbates DSS-induced colitis susceptibility by impairing TLR4/NF-κB signaling, thereby inducing neutrophil necroptosis [53]. The protein tyrosine phosphatase non-receptor type 2 (PTPN2) phosphatase strengthens junctions between macrophages and epithelial cells to maintain intestinal barrier function [54]. In diabetic injury, dexmedetomidine promotes anti-inflammatory macrophage polarization through the matrix metallopeptidase 23B (MMP23B) pathway to enhance intestinal barrier function and to reduce mitochondrial dysfunction [55]. Additionally, Cystathionine gamma-lyase (Cth) protein enhances efferocytosis via ERK1/2 activation in aging-related enteropathy, and thus promote intestinal barrier repair [56]. Astragaloside IV induces anti-inflammatory macrophage polarization via AKT signaling in radiation enteritis to decrease inflammation response [57] (Table 1). *Lactobacillus murinus* EVs activate anti-inflammatory macrophage polarization via TLR2 to repair Deoxynivalenol (DON)-induced injury [58,59]. Saikosaponin A blocks the CH25H/25-OHC-NLRP3 pathway to inhibit inflammation in macrophages [60], while saponin VI reprograms macrophage polarization by modulating the autophagy-NLRP3 circuit, shifting the balance from pro-inflammatory macrophage toward anti-inflammatory macrophage phenotypes [61]. In biliary diseases, excess LPS overstimulates the TLR4/MyD88/NF-κB axis, while the intestinal microbiome exacerbates intestinal leakage by promoting cholestasis-mediated cell death and inflammation through inflammasome activation in macrophages [44] (Table 2). In IBD, TLR4/NF-κB hyperactivation drives pro-inflammatory macrophage polarization, disrupting tight junctions via TNF-α/IL-6 [62]. Moreover, Mcpip1 deficiency specifically in macrophages promotes a pro-inflammatory phenotype, halts maturation, and exacerbates intestinal inflammation in a manner dependent on the Atf3-Ap1s2 pathway [63] (Table 1).

**Table 1:** Core Roles of Macrophages in Intestinal Barrier Function.

Function Direction	Key Macrophage Subset/Type	Core Mechanism/Effector Molecule	Main Function/Outcome	References
Barrier Disruption	Pro-inflammatory macrophage	TNF-α, IL-1β (via NOX4/ROS pathway)	Directly degrade tight junction proteins	
		CCL3	Recruit neutrophils and exacerbate inflammation	
	Claudin-2 (epithelial expression)	Forms abnormal pores	Increases intestinal permeability, facilitates microbiota translocation, and promotes systemic inflammation	
	TLR4/NF-κB pathway activation (e.g., by LPS)	TLR4-MyD88 signaling leading to NF-κB activation	Sustains macrophage activation, expands intestinal leakage, and forms a microbiota–macrophage vicious circle	[44,45]
	Dysfunction in IBD	Overactivation of IL-23/IL-17 pathway	Exacerbates barrier disruption and chronic inflammation	[17,46]
Barrier Protection	LGR5^+^	Secrete Wnt3a/R-spondin	Activate crypt stem cells and drive crypt regeneration	[33]
	IL-10-dependent subsets	Secrete IL-10 (sensing TLR2/4 signals)	Inhibit hyperinflammation and maintain epithelial tight junction integrity	[43]
	Anti-inflammatory macrophage	Secrete IL-10	Activate epithelial JAK1/STAT3 pathway and promote tight junction protein synthesis	[49,50,51]
	CD11c^+^	TGF-β, Retinoic acid	Induce Treg differentiation and prevent abnormal responses to commensal bacteria	[48]
	LACC1	Key molecule	Enhance macrophage bacterial clearance and reduce microbiota translocation	[51]
	Heme-induced anti-inflammatory macrophage	HO-1-dependent and -independent pathways	Repair barrier	[50]
	Probiotics (e.g., *L. paracasei*) & EPS	Balance Th17/Treg cells	Promote anti-inflammatory macrophage polarization and enhance mucus layer and tight junctions	[49]
	GM-CSF	Maintain macrophage homeostasis	Support epithelial repair	[52]

### 6.2. Metabolic Crosstalk Critically Maintains Homeostasis

The metabolic crosstalk between the gut microbiota, their metabolites, and intestinal macrophages is a cornerstone of intestinal barrier homeostasis. This crosstalk may reinforce an anti-inflammatory tone in macrophages, which is essential for barrier repair and maintenance. Butyrate stands as a prime example of this beneficial role. Under homeostatic conditions, it is well-established that butyrate promotes mucosal healing by enhancing macrophage-derived WNT/ERK signaling, thereby upregulating goblet cell markers MUC2 and SPDEF to strengthen the mucus layer [64]. This paradigm of metabolite-driven immunoregulation is further supported by other microbial signals. For instance, cathepsin K (CTSK) from the microbiota can induce TLR4-dependent anti-inflammatory macrophages [13,65], and SCFA-producing bacteria collectively suppress JAK/STAT3/FOXO3 signaling to ameliorate colitis [66]. The propionic acid produced by Lactobacillus johnsonii operates through a similar principle, attenuating colitis by inhibiting pro-inflammatory macrophage polarization via MAPK modulation [67]. However, emerging research nuances this simplistic ‘good metabolite’ narrative by revealing context-dependent immunomodulation, particularly under inflammatory conditions. Butyrate itself can exhibit a dual nature: while beneficial at homeostasis, it has been shown to contribute to inflammatory responses during TLR activation by inducing NLRP3 inflammasome formation through GPR43 [64]. This shift underscores that the metabolic crosstalk can be disrupted or altered in disease states. For example, high-fat diets that deplete butyrate-producing bacteria lead to dysfunctional iron metabolism in macrophages, propagating anemia and inflammation [68,69]. Therapeutically, strategies aimed at restoring this beneficial crosstalk show great promise. Exogenous agents like a butyrate-melatonin complex can halt colitis progression by dual modulation of the microbiome and the NLRP3/caspase-1 pathway, effectively re-educating macrophages towards an anti-inflammatory phenotype [70,71,72]. Similarly, metformin may suppress TLR4/NF-κB signaling and synergizes with SCFAs to reinforce anti-inflammatory macrophage differentiation [58,59]. Conversely, genetic disruptions such as PTPN2 deficiency, which impairs IL-10 signaling and tight junction integrity, highlight critical nodes in this network that can be targeted for rescue [54]. For a systematic overview, Table 2 summarizes the roles of major metabolites and modulators in macrophage–epithelial metabolic crosstalk and their effects on barrier function.

**Table 2:** Key Signaling Pathways and Metabolic Crosstalk Regulating Macrophage Function to Influence the Intestinal Barrier.

Regulatory Type/Factor	Key Target/Pathway/Molecule	Regulatory Mechanism/Effect	Main Function/Outcome	References
Signaling Pathway Regulation	Tim-3 (macrophages)	Tim-3 deficiency impairs TLR4/NF-κB signaling and induces neutrophil necroptosis	Exacerbates DSS-induced colitis susceptibility	[53]
	PTPN2 (Protein tyrosine phosphatase non-receptor type 2)	Strengthens junctions between macrophages and epithelial cells	Maintains intestinal barrier function; Deficiency disrupts IL-10 signaling and degrades tight junction proteins; Activation restores metabolic crosstalk	[54]
	MMP23B (Matrix metallopeptidase 23B)	Dexmedetomidine promotes anti-inflammatory macrophage polarization	Enhances intestinal barrier function and reduces mitochondrial dysfunction in diabetic injury	[55]
	Cth (Cystathionine gamma-lyase)	Enhances efferocytosis via ERK1/2 activation	Promotes intestinal barrier repair in aging-related enteropathy	[56]
	AKT signaling	Astragaloside IV induces anti-inflammatory macrophage polarization	Decreases inflammatory response in radiation enteritis	[57]
	TLR2	*Lactobacillus murinus* EVs activate anti-inflammatory macrophage polarization	Repair Deoxynivalenol (DON)-induced injury	[58,59]
	CH25H/25-OHC-NLRP3 pathway	Saikosaponin A blocks this pathway	Inhibits macrophage inflammation	[60]
	Autophagy-NLRP3 circuit	Saponin VI reprograms macrophage polarization by modulating this circuit	Shifts macrophage polarization balance from pro-inflammatory macrophage toward anti-inflammatory macrophage phenotypes	[61]
	TLR4/MyD88/NF-κB axis	Excess LPS overstimulation; Microbiome promotes cholestasis-mediated cell death and inflammation via inflammasome activation in macrophages in biliary disease	Exacerbates intestinal leakage	[44]
	TLR4/NF-κB (IBD)	Hyperactivation drives pro-inflammatory macrophage polarization	Disrupts tight junctions via TNF-α/IL-6	[63]
	Mcpip1 (macrophage-specific deficiency)	Dependent on Atf3-Ap1s2 pathway, promotes pro-inflammatory macrophage phenotype, and halts maturation	Exacerbates intestinal inflammation	[63]
Metabolic Crosstalk (Core)	Butyrate	Activates macrophage/WNT/ERK signaling pathway	Promotes intestinal mucus barrier repair	
		Synergizes with butyrate-primed anti-inflammatory macrophages	Induces significantly higher expression of goblet cell markers (MUC2, SPDEF)	
		Under TLR activation, triggers NLRP3 inflammasome formation via GPR43		[64]
	High-fat diet (HFD)	Downregulates butyrate-producing bacteria, impairing macrophage-mediated iron export	Fuels cycles of anemia and inflammation	[68,69]
	SCFA-producing bacteria	Suppresses JAK/STAT3/FOXO3 axis and drives anti-inflammatory macrophage polarization	Attenuates experimental ulcerative colitis	[66]
	Propionic acid (from *L. johnsonii*)	Suppresses pro-inflammatory macrophage polarization through MAPK pathway modulation	Alleviates colitis	[67]
	Butyrate-melatonin complex	Dual modulation of microbiome and NLRP3/caspase-1 inflammatory pathways, redirecting macrophage polarization	Halts ulcerative colitis progression in mice	[70]
	Microbial metabolite reprogramming	Reprograms macrophage metabolism (e.g., enhancing oxidative phosphorylation) and inhibits pro-inflammatory responses		[71,72]
	Metformin	Inhibits TLR4/NF-κB signaling (reducing TNF-α, IL-6), enhances oxidative phosphorylation metabolism promoting anti-inflammatory macrophage differentiation, synergizes with elevated intestinal SCFAs	Collectively improves intestinal barrier function	[58,59]
	Cathepsin K (CTSK)	Microbiota-stimulated secretion drives TLR4-dependent anti-inflammatory macrophage polarization	Promotes metastatic progression in colorectal cancer	[13,65]
	PTPN2/IL-10 (Metabolic Crosstalk)	PTPN2 deficiency in macrophages disrupts IL-10 signaling and leads to degradation of epithelial tight junction proteins; Specific PTPN2 activation or exogenous IL-10 restores metabolic crosstalk	Restores metabolic crosstalk between macrophages and intestinal epithelial cells, thereby ameliorating barrier damage	[54]

## 7. Disease

### 7.1. Inflammatory Bowel Disease (IBD)

Dysfunctional intestinal macrophages are a key driver of pathology in IBD. Embryo-derived macrophages (EMFs) and monocyte-derived macrophages (MoMFs) exhibit functional imbalance in IBD: EMFs maintain homeostasis, while MoMFs promote inflammation [73]. Embryo-derived resident macrophages lose their anti-inflammatory properties in the IBD environment. Concurrently, circulating monocytes are extensively recruited to the inflamed intestine. Under the stimulation of the local microenvironment (characterized by factors such as high levels of IFN-γ, GM-CSF, LPS, and TLR signaling activated by dysbiosis), these monocytes aberrantly differentiate into pro-inflammatory macrophages. These activated macrophages exhibit heightened expression and release large quantities of pro-inflammatory cytokines, including TNF-α, IL-1β, IL-6, IL-12, and IL-23 (Table 1). This ‘pro-inflammatory cytokine storm’ is pivotal in initiating and sustaining intestinal inflammation. Macrophage phenotypic polarization—for example, switching from pro-inflammatory macrophage to anti-inflammatory macrophage—is a core mechanism regulating intestinal barrier balance. Certain intestinal metabolites influence the intestinal barrier by modulating macrophage polarization: β-hydroxybutyrate promotes anti-inflammatory macrophage polarization via the STAT6 signaling pathway, alleviating colitis [74]. Intestinal microbiota dysbiosis can activate macrophage TLR signaling, while targeted nanoparticles regulate macrophage polarization and reduce inflammation by inhibiting endosomal TLR signaling [75]. The TLR9 agonist Cobitolimod exerts its therapeutic effects in ulcerative colitis patients by inducing IL-10-producing wound-healing macrophages and regulatory T cells [76]. Pro-inflammatory cytokines released by macrophages, particularly TNF-α, directly damage intestinal epithelial cells and disrupt tight junction proteins between them, significantly increasing intestinal permeability [77]. Sargramostim (recombinant human GM-CSF) is used in the treatment of active Crohn’s disease by promoting the generation and function of macrophages and granulocytes, thereby enhancing mucosal immunity and repair [78]. Furthermore, matrix metalloproteinases (MMPs) secreted by macrophages (such as MMP-12) facilitate the transmigration of macrophages themselves across the epithelial layer, further compromising tight junction integrity [79] and exacerbating barrier damage. The loss of barrier function allows commensal bacteria and antigens to translocate into the intestinal lamina propria, perpetuating immune system activation and establishing a vicious cycle. Sustained inflammation induces the release of MMPs and reactive oxygen species (ROS), leading to tissue ulceration and fibrosis. ROS regulate the development of the macrophage-microbiome interface, impacting early intestinal homeostasis [80].

Furthermore, intestinal macrophages are essential components of the ISC niche, providing critical signals that maintain epithelial homeostasis and drive regeneration after injury. Dysfunctional intestinal macrophages impair the regenerative capacity of intestinal stem cells (ISCs) through multiple mechanisms in IBD, aggravating mucosal injury [81]. In IBD, sustained inflammation disrupts macrophage function, leading to an accumulation of pro-inflammatory macrophages [82]. Inhibition of Wnt/β-catenin signaling: Macrophage-derived EDA-A2 protein suppresses β-catenin activity via the miR-494/EDA2R axis, blocking Wnt signaling-mediated ISC self-renewal and differentiation [77]. Disruption of niche support: Inflammatory cytokines such as IFN-γ damage Paneth cells and stromal cells surrounding the crypts, weakening the Wnt/Notch support signals crucial for ISCs. Collectively, these mechanisms lead to delayed epithelial repair, persistent ulceration, and progression of fibrosis [83].

Targeting metabolic crosstalk presents a promising yet challenging therapeutic strategy for IBD. Several metabolites and compounds have shown potential in preclinical models, though their clinical translatability requires rigorous validation. For instance, the ketone body β-hydroxybutyrate was found to enhance the anti-inflammatory effects of macrophages via STAT6 in experimental settings [74], suggesting a potential therapeutic avenue worth further exploration. Similarly, preclinical studies indicate that compounds like Polyphyllin VI and HMB can regulate macrophage polarization through specific pathways such as the autophagy-NLRP3 axis and ERK/NF-κB pathway to alleviate colitis [61,84], highlighting these mechanisms as potential intervention targets. The well-established effect of butyrate in suppressing NF-κB activation in macrophages from UC patients provides a stronger mechanistic rationale for SCFA-based therapies [85]. Notably, existing drugs such as mesalazine (5-ASA) already exert part of their effect by modulating core inflammatory pathways like NF-κB in macrophages, validating this approach clinically. However, the field faces significant challenges. The pro-inflammatory role of epigenetic regulators like METTL3, demonstrated in mechanistic studies [86], points to a novel target, but developing specific and safe inhibitors remains a formidable task. While dietary interventions targeting TRPM8 can reprogram macrophage metabolism and reduce colitis in models [87], their efficacy and practicality in diverse patient populations need confirmation in controlled trials. Furthermore, observations such as GIP linking high-energy diets to increased MCP-1 levels reveal plausible mechanisms for diet-induced inflammation [85], but this association necessitates further investigation to establish causality in humans. In summary, while preclinical evidence robustly identifies numerous components of metabolic crosstalk as potential therapeutic targets, the translation of these findings into clinical practice hinges on future research employing larger sample sizes and more robust clinical trial designs to confirm their efficacy and safety in IBD patients.

### 7.2. Infection and Sepsis

Models of infection and sepsis reveal a complex network of interactions. In Salmonella infection, crypt macrophages precisely regulate epithelial NF-κB activity through TNF signaling, balancing antimicrobial defense and barrier protection [88]. In neonatal necrotizing enterocolitis (NEC), CCL3 chemokines recruit pro-inflammatory macrophages to damage intestinal epithelial cells through NLRP3 inflammasomes [89]. In a stress model, bind stress activates mesenteric macrophages and perturbs microbiota, resulting in intestinal leakage [90]. In hypertensive rats, monoglyceride laurate relieves colitis by inhibiting macrophage infiltration [91]. In sepsis, macrophages regulate intestinal barrier function through multiple pathways. Neutrophil extracellular traps (NETs) push macrophages to a pro-inflammatory macrophage phenotype through TGF-β signaling, disrupting tight junction [92].In response to these crises, HMGB1 inhibitors have a two-pronged approach in sepsis models: both inhibition of macrophage NETosis and blockade of pro-inflammatory macrophage-polarized [93].

Macrophage polarization phenotypes are critical in regulating intestinal injury: Astragaloside drives macrophage polarization towards the anti-inflammatory phenotype by reshaping intestinal microbiota and short-chain fatty acids [94]; the Ghrelin/GHSR axis induces anti-inflammatory macrophages and alleviates intestinal barrier dysfunction by inactivating E2F1/NF-κB signaling. Tetrahedral framework nucleic acid alleviates sepsis-induced intestinal injury by regulating anti-inflammatory macrophages [95]. Furthermore, in the inflammasome pathway, Card9 exerts protective effects by regulating Ripk2-mediated activation of the NLRP3 inflammasome [96], while systemic interventions such as P2X7 receptor blockade [97] and engineered capsules [98] also mitigate barrier disruption by regulating macrophage-associated inflammation. Conversely, diabetes promotes pro-inflammatory macrophage polarization via miR-3061/Snail1 signaling, exacerbating intestinal injury [99].

Moreover, macrophage depletion disrupts microbial homeostasis, leading to intestinal fungal overgrowth and invasion into the blood and intestinal wall, exacerbating systemic infection [100]. Intestinal macrophages uphold homeostasis by phagocytosing microbes, secreting cytokines to activate antifungal immunity, and clearing apoptotic cells to maintain barrier integrity. Their depletion disrupts these mechanisms, permitting Candida spp. to transition into invasive pathogens. Barrier breakdown and immune dysregulation foster fungal proliferation [101]. IBD patients, particularly post-colectomy, show elevated risks of invasive candidiasis, linked to defective macrophage function and impaired barrier repair [102]. Concurrently, macrophage depletion alters intestinal microbiota composition through fungal proliferation, increasing the risk of bacterial translocation [103], while Candida albicans synergizes with Klebsiella pneumoniae to worsen sepsis via intestinal dysbiosis and enhanced inflammation [104]. Notably, while macrophage elimination elevates bacterial translocation and subsequent intestinal-origin septicemia risk, it paradoxically mitigates symptoms and mortality by curbing excessive inflammation [105], highlighting the importance of functional balance.

Collectively, macrophages constitute the core network of intestinal barrier defense in sepsis by regulating microbiota balance, polarization phenotypes, and inflammatory pathways.

### 7.3. Intestinal Tumor

In intestinal tumor development, macrophages and the intestinal barrier engage in tightly coupled bidirectional regulation. Tumor-associated macrophages (TAMs) critically influence tumor progression through angiogenesis, invasion, metastasis, and immunosuppression. While early-stage tumors are predominantly countered by anti-tumor pro-inflammatory macrophages, disease advancement drives their polarization toward pro-tumorigenic anti-inflammatory TAMs. Notably, LPS amplifies pro-inflammatory macrophage polarization and the release of pro-inflammatory mediators (IL-1β, IL-6, TNF-α), while anti-inflammatory TAMs facilitate tumor growth via secretion of survival factors.

Central to this process, the microbiota–immune axis regulates tumor initiation through intestinal barrier compromise (“leaky intestinal”). This breach enables microbial metabolites such as D-lactate and bacterial products to activate macrophage TLR4/NF-κB signaling, triggering chronic inflammation and DNA-damaging cytokines including TNF-α and IL-6; concurrently, high-fat diets impair barrier function by disrupting butyrate metabolism [68,106]. Second, persistent stimulation induces macrophage polarization toward pro-metastatic anti-inflammatory phenotypes: microbiota-activated cathepsin K accelerates anti-inflammatory differentiation via the TLR4 pathway [65], while D-lactate enhances anti-inflammatory TAM polarization by stabilizing HIF-1α [107]. These anti-inflammatory TAMs secrete IL-10 and TGF-β and express PD-L1, directly suppressing T/NK cell activity and creating an immune-evasive microenvironment.

Furthermore, TAMs secrete MMP-9, which directly degrades intestinal epithelial tight junction proteins such as ZO-1, Occludin, and Claudin-1, compromising barrier integrity, and VEGF, which promotes angiogenesis. This dual action accelerates tumor invasion and metastasis; crucially, this destructive process can be counteracted by barrier-restoring agents. For example, ENPP2 inhibitors enhance tight junction repair by upregulating epithelial Occludin/Claudin-1 expression while blocking macrophage TLR4/NF-κB signaling to reduce inflammation [108]. Tetrahydrocurcumin strengthens ZO-1 expression and inhibits infiltration of Arg-1^+^/CD206^+^ anti-inflammatory macrophage TAMs by suppressing the SPP1/CD44 axis [107]. Senegalia polysaccharides preserve barrier function through inhibition of the NF-κB/MLCK pathway in macrophages [109]. Metabolic intervention strategies, including the Jianpi Jiedu Formula (tryptophan metabolism-AhR signaling) [71], dioscin (PPARγ/STAT6 pathway) [110], FXR agonists [111], and microbiota-reprogramming Chinese herbs [112,113], all effectively inhibit anti-inflammatory TAM polarization.

The interplay between macrophages and the intestinal barrier also leads to therapy resistance. Anti-inflammatory TAMs undermine the efficacy of radiotherapy and chemotherapy by shielding cancer stem cells, while microbial imbalance can suppress the response to immunotherapy. However, this detrimental process can be reversed through targeted interventions, thereby restoring treatment sensitivity. For example, Lactobacillus casei combined with L. reuteri promotes macrophage pro-inflammatory polarization through TLR4 inhibition [114]; GM-CSF corrects microbial imbalance to suppress hepatic macrophage TLR4/IL-6 signaling [115].

Collectively, these findings establish the intestinal barrier–macrophage axis as a master regulatory network in tumor progression. Barrier restoration counteracts pro-tumor TAM phenotypes, revealing critical therapeutic targets.

## 8. Concluding Remarks

As the body’s primary defense against pathogens and guardian of internal stability, the intestinal barrier depends on the coordinated interplay of epithelial cells—including intestinal epithelial cells, goblet cells, and Paneth cells—alongside macrophages residing within the lamina propria [116]. In this review, we systematically analyze the interaction network between intestinal barrier cells and innate macrophages, and analyze their dual roles in homeostasis maintenance and disease pathogenesis.

The breakdown in communication between gut macrophages and the intestinal barrier lies at the heart of several bowel diseases—from IBD and infections to colorectal cancer. When the barrier weakens, microbes and their components slip through the lining, stirring macrophages into action and sparking inflammation. But when these immune cells themselves malfunction, whether by losing their ability to calm inflammation or being stuck in attack mode, they make things worse: they disrupt healing, damage the barrier further, and lock the system into a destructive cycle.

That is why treatment strategies are beginning to look beyond simply fighting inflammation. The new focus is on restoring the natural dialogue between macrophages and barrier cells—helping them work together again. The goal is not to shut down immunity, but to rebalance the intestinal environment so these cells can communicate clearly and support real repair over the long term. Getting there will take more than immunology—it will require teaming up with microbiology, materials science, and neurobiology to build nuanced, personalized approaches that truly reset the conversation in the gut. In the future, it will be necessary to integrate single-cell transcriptome, spatial multiomics and organoid co-culture technologies to analyze the spatiotemporal heterogeneity of macrophage subsets. At the same time, the development of tissue-specific delivery systems or gene-edited macrophages may break through existing therapeutic bottlenecks. Through interdisciplinary collaboration and technological innovation, therapeutic strategies targeting the intestinal cell–macrophage interaction network are expected to provide more precise solutions for diseases such as IBD, sepsis, and colon cancer.

## Data Availability

No new data were created or analyzed in this study.

## Abbreviations

The following abbreviations are used in this manuscript:

IBD	Inflammatory Bowel Disease
IECs	Intestinal Epithelial Cells
ZO-1	Zona Occludens 1
GLP-2	Glucagon-Like Peptide 2
SCFAs	Short-Chain Fatty Acids
GALT	Gut-Associated Lymphoid Tissue
SUCNR1	Succinate Receptor Antagonist 1
IELs	Intraepithelial Lymphocytes
sIgA	Secretory Immunoglobulin A
FXR	Farnesoid X Receptor
MUC2	Mucin2
EMPs	Erythro-Myeloid Progenitors
HSCs	Hematopoietic Stem Cells
P2X7	Purinergic 2X7
AhR	Aryl Hydrocarbon Receptor
GPR109A	G Protein-Coupled Receptor 109 A
MDP	Muramyl Dipeptide
NOD2	Nucleotide-Binding Oligomerization Domain 2
PTPN2	Protein Tyrosine Phosphatase Non-Receptor Type 2
MMP23B	Matrix Metallopeptidase 23B
Cth	Cystathionine Gamma-Lyase
DON	Deoxynivalenol
SPDEF	Sam Pointed Domain Containing Ets Transcription Factor
CTSK	Cathepsin K
EMFs	Embryo-Derived Macrophages
MoMFs	Monocyte-Derived Macrophages
MMPs	Matrix Metalloproteinases
ROS	Reactive Oxygen Species
ISCs	Intestinal Stem Cells
NEC	Neonatal Necrotizing Enterocolitis
TAMs	Tumor-Associated Macrophages
TLR2	Toll Like Receptor 2
